# Supplementary material for: Evaluation of Midwives’ Practises on Herpetic Infections during Pregnancy: A French Vignette-Based Study
Source: Healthcare (Basel). 2023 Jan 28;11(3):364. doi: 10.3390/healthcare11030364 (PMC9914294; doi:10.3390/healthcare11030364)
Supplement: Supplementary file 1 [file healthcare-11-00364-s001.zip › healthcare-2141276-supplementary.pdf]

Table S1: Checklist for Reporting Results of Internet E-Surveys (CHERRIES)

Checklist Item	***Explanation***	***Page Number***
Describe survey design	Describe target population, sample frame. Is the sample a convenience sample? (In "open" surveys this is most likely.)	2
IRB approval	Mention whether the study has been approved by an IRB.	2
Informed consent	Describe the informed consent process. Where were the participants told the length of time of the survey, which data were stored and where and for how long, who the investigator was, and the purpose of the study?	2
Data protection	If any personal information was collected or stored, describe what mechanisms were used to protect unauthorized access.	NA
Development and testing	State how the survey was developed, including whether the usability and technical functionality of the electronic questionnaire had been tested before fielding the questionnaire.	2-3
Open survey versus closed survey	An "open survey" is a survey open for each visitor of a site, while a closed survey is only open to a sample which the investigator knows (password-protected survey).	2
Contact mode	Indicate whether or not the initial contact with the potential participants was made on the Internet. (Investigators may also send out questionnaires by mail and allow for Web-based data entry.)	2
Advertising the survey	How/where was the survey announced or advertised? Some examples are offline media (newspapers), or online (mailing lists – If yes, which ones?) or banner ads (Where were these banner ads posted and what did they look like?). It is important to know the wording of the announcement as it will heavily influence who chooses to participate. Ideally the survey announcement should be published as an appendix.	2
Web/E-mail	State the type of e-survey (eg, one posted on a Web site, or one sent out through e-mail). If it is an e-mail survey, were the responses entered manually into a database, or was there an automatic method for capturing responses?	2
Context	Describe the Web site (for mailing list/newsgroup) in which the survey was posted. What is the Web site about, who is visiting it, what are visitors normally looking for? Discuss to what degree the content of the Web site could pre-select the sample or influence the results. For example, a survey about vaccination on a anti-immunization Web site will have different results from a Web survey conducted on a government Web site	2
Mandatory/voluntary	Was it a mandatory survey to be filled in by every visitor who wanted to enter the Web site, or was it a voluntary survey?	2
Incentives	Were any incentives offered (eg, monetary, prizes, or non-monetary incentives such as an offer to provide the survey results)?	2
Time/Date	In what timeframe were the data collected?	2

Randomization of items or questionnaires	To prevent biases items can be randomized or alternated.	3
Adaptive questioning	Use adaptive questioning (certain items, or only conditionally displayed based on responses to other items) to reduce number and complexity of the questions.	3
Number of Items	What was the number of questionnaire items per page? The number of items is an important factor for the completion rate.	NA
Number of screens (pages)	Over how many pages was the questionnaire distributed? The number of items is an important factor for the completion rate.	NA
Completeness check	It is technically possible to do consistency or completeness checks before the questionnaire is submitted. Was this done, and if "yes", how (usually JAVAScript)? An alternative is to check for completeness after the questionnaire has been submitted (and highlight mandatory items). If this has been done, it should be reported. All items should provide a non-response option such as "not applicable" or "rather not say", and selection of one response option should be enforced.	3
Review step	State whether respondents were able to review and change their answers (eg, through a Back button or a Review step which displays a summary of the responses and asks the respondents if they are correct).	3
Unique site visitor	If you provide view rates or participation rates, you need to define how you determined a unique visitor. There are different techniques available, based on IP addresses or cookies or both.	2
View rate (Ratio of unique survey visitors/unique site visitors)	Requires counting unique visitors to the first page of the survey, divided by the number of unique site visitors (not page views!). It is not unusual to have view rates of less than 0.1 % if the survey is voluntary.	NA
Participation rate (Ratio of unique visitors who agreed to participate/unique first survey page visitors)	Count the unique number of people who filled in the first survey page (or agreed to participate, for example by checking a checkbox), divided by visitors who visit the first page of the survey (or the informed consents page, if present). This can also be called "recruitment" rate.	3
Completion rate (Ratio of users who finished the survey/users who agreed to participate)	The number of people submitting the last questionnaire page, divided by the number of people who agreed to participate (or submitted the first survey page). This is only relevant if there is a separate "informed consent" page or if the survey goes over several pages. This is a measure for attrition. Note that "completion" can involve leaving questionnaire items blank. This is not a measure for how completely questionnaires were filled in. (If you need a measure for this, use the word "completeness rate".)	3

Cookies used	Indicate whether cookies were used to assign a unique user identifier to each client computer. If so, mention the page on which the cookie was set and read, and how long the cookie was valid. Were duplicate entries avoided by preventing users access to the survey twice; or were duplicate database entries having the same user ID eliminated before analysis? In the latter case, which entries were kept for analysis (eg, the first entry or the most recent)?	3
IP check	Indicate whether the IP address of the client computer was used to identify potential duplicate entries from the same user. If so, mention the period of time for which no two entries from the same IP address were allowed (eg, 24 hours). Were duplicate entries avoided by preventing users with the same IP address access to the survey twice; or were duplicate database entries having the same IP address within a given period of time eliminated before analysis? If the latter, which entries were kept for analysis (eg, the first entry or the most recent)?	3
Log file analysis	Indicate whether other techniques to analyze the log file for identification of multiple entries were used. If so, please describe.	3
Registration	In "closed" (non-open) surveys, users need to login first and it is easier to prevent duplicate entries from the same user. Describe how this was done. For example, was the survey never displayed a second time once the user had filled it in, or was the username stored together with the survey results and later eliminated? If the latter, which entries were kept for analysis (eg, the first entry or the most recent)?	NA
Handling of incomplete questionnaires	Were only completed questionnaires analyzed? Were questionnaires which terminated early (where, for example, users did not go through all questionnaire pages) also analyzed?	3
Questionnaires submitted with an atypical timestamp	Some investigators may measure the time people needed to fill in a questionnaire and exclude questionnaires that were submitted too soon. Specify the timeframe that was used as a cut-off point, and describe how this point was determined.	NA
Statistical correction	Indicate whether any methods such as weighting of items or propensity scores have been used to adjust for the non-representative sample; if so, please describe the methods.	NA

This checklist has been modified from Eysenbach G. Improving the quality of Web surveys: the Checklist for Reporting Results of Internet E-Surveys (CHERRIES). J Med Internet Res. 2004 Sep 29;6(3):e34 [erratum in J Med Internet Res. 2012; 14(1): e8.]. Article available at https://www.jmir.org/2004/3/e34/; erratum available https://www.jmir.org/2012/1/e8/. Copyright ©Gunther Eysenbach. Originally published in the Journal of Medical Internet Research, 29.9.2004 and 04.01.2012.

This is an open-access article distributed under the terms of the Creative Commons Attribution License (https://creativecommons.org/licenses/by/2.0/), which permits unrestricted use, distribution, and reproduction in any medium, provided the original work, first published in the Journal of Medical Internet Research, is properly cited.

Survey S1: Questionnaire of the study

Socio-demographic data

1) You are?

a. A woman

b. A man

2) How old are you?

3) How many years have you been working as a registered midwife?

4) What is your main mode of practice?

If you have a mixed practice (half-time hospital and half-time private practice), please check the practice in which you have practiced the longest

A : Non hospital midwive

C : hospital midwive in a maternity hospital with <1000 births per year

D : hospital midwive in a maternity hospital with 1000 to 3000 births per year

E: hospital midwive in a maternity hospital with >3000 births per year

5) In which province do you work?

Practices and management of herpes infections in pregnant women and newborns

Some questions are single choice and others are multiple choice

Clinical vignette #1 (questions 6-9):

A 30-year-old nulliparous woman comes to the emergency room with vulvar pain that has been present for several days. She has no other symptoms and her pregnancy is proceeding normally. She is at the end of 25 weeks of gestation today.

On clinical check-up, you find clusters of painful and inflammatory vesicles on the vulva and vagina, typical of a herpetic infection. You ask the patient about her history and she tells you that she has never had these symptoms before.

You have performed para-clinical exams and the laboratory gives you the results:

PCR HSV 1 + / PCR HSV 2 - /IgG HSV1 - / IgG HSV2 -

6) What do you think is the most probable diagnosis? (*: correct answer)

a) Initial primary infection*	n=693 (95.2%)
b) Initial non-primary infection	n=20 (2.7%)
c) Recurrence	n=2 (0.3%)
d) I don't know	n=13 (1.8%)

7) What is, in your opinion, the appropriate treatment to adopt for this pregnancy? (*: correct answer)

- No treatment	n=3 (0.4)
- Prophylactic antiviral treatment at 36 weeks of gestation	n=425 (58.4)*
- Therapeutic antiviral treatment	n=692 (95.1)*

7bis) What is, in your opinion, the appropriate management to adopt for this pregnancy? (*: correct answer)

- Programmed cesarean section at term	n=19 (2.6)
- vaginal delivery at term if no new herpetic manifestations	n=622 (85.4)*
- Don't know	n=87 (11.9)

After appropriate management and a pregnancy without recurrence of herpes, the parents are anxious about the risk to their child associated with neonatal herpes. They talk to you and discuss the herpetic episode at 25 days' gestation.

8) What can you say about the risk of neonatal herpes in this asymptomatic newborn? (*: correct answer)

a) High risk situation (>20% risk of developing neonatal herpes)	n=19 (2.6)
b) Low risk situation (<20% risk of developing neonatal herpes)	n=319 (43.8)*
c) No risk situation (<0.1% risk of developing neonatal herpes)	n=295 (40.5)*
d) Don't know	n=95 (13.1)

9) Can the patient breastfeed her child? (*: correct answer)

a) Yes	n=727 (99.9)*
b) No	n=1 (0.1)

Clinical vignette n°2 (question 10 to 13):

A 30-year-old nulliparous woman comes to the emergency room with vulvar pain for several days. She has no other symptoms and her pregnancy is proceeding normally. She is at 38 weeks of gestation today.

On clinical examination, you find clusters of painful and inflammatory vesicles on the vulva and vagina, typical of a herpetic infection. You ask the patient about her history and she tells you that she has regularly had pimples for several years.

You have performed para-clinical examinations and the laboratory gives you the results:

PCR HSV1+ / PCR HSV2 - / IgG HSV1- / IgG HSV 2 +

10) What do you think is the most likely diagnosis? (*: correct answer)

a) Initial primary infection	n=110 (15.1)
b) Initial non-primary infection	n=345 (47.4)*
c) Recurrence	n=246 (33.8)
d) Don't know	n=27 (3.7)

11) What is, in your opinion, the appropriate treatment to adopt for this pregnancy? (*: correct answer)

- No treatment	n=2 (0.3)
- Prophylactic antiviral treatment at 36 weeks of gestation	n=363 (49.9)
- Therapeutic antiviral treatment	n=500 (68.8)*

11bis) What is, in your opinion, the appropriate management to adopt for this pregnancy? (*: correct answer)

- Programmed cesarean section at term	n=257 (35.3)*
- vaginal delivery this day	n=5 (0.7)
- Don't know	n=466 (64.0)

In hospital, the parents are anxious about the risk to their child associated with neonatal herpes. They talk to you and discuss the herpes episode of a few days ago.

12) What can you say about the risk of neonatal herpes in this asymptomatic newborn? (*: correct answer)

a) High risk situation (>20% risk of developing neonatal herpes)	n=290 (39.8)*
b) Low risk situation (<20% risk of developing neonatal herpes)	n=268 (36.8)
c) No risk situation (<0.1% risk of developing neonatal herpes)	n=45 (6.2)
d) Don't know	n=125 (17.2)

13) Can the patient breastfeed her child? (*: correct answer)

a) Yes	n=714 (98.1)*
b) No	n=14 (1.9)

Clinical vignette #3 (questions 14-17):

A 30-year-old nulliparous woman comes to you for her pregnancy consultation. She does not describe any symptoms and her pregnancy is proceeding normally. She is at 38 weeks of amenorrhea on this day.

On clinical examination, you find clusters of vesicles on the vulva and vagina, typical of a herpes infection. However, these vesicles are not felt by the patient. You ask the patient about her history and she tells you that she has had these pimples regularly for several years.

You have performed para-clinical tests and the laboratory gives you the results:

PCR HSV1 + / PCR HSV2 - / IgG HSV1 + / IgG HSV2 -

14) What do you think is the most likely diagnosis? (*: correct answer)

a) Initial primary infection	n=4 (0.5)
b) Initial non-primary infection	n=52 (7.1)
c) Recurrence	n=659 (90.5)*
d) Don't know	n=13 (1.8)

15) What do you think is the appropriate treatment for this pregnancy? (*: correct answer)

- No treatment	n=16 (2.2)
- Prophylactic antiviral treatment at 36 weeks of gestation	n=442 (60.7)
- Therapeutic antiviral treatment	n=409 (56.1)*

15bis) What do you think is the appropriate management for this pregnancy? (*: correct answer)

- Programmed cesarean section at term	n=161 (22.1)*
- vaginal delivery this day	n=15 (2.1)*
- don't know	n=552 (75.8)

While in the hospital, the parents are anxious about the risk to their child of neonatal herpes. They talk to you and discuss the herpes episode of a few days ago.

15) What can you say about the risk of neonatal herpes in this asymptomatic newborn? (*: correct answer)

a) High risk situation (>20% risk of developing neonatal herpes)	n=129 (17.7)
b) Low risk situation (<20% risk of developing neonatal herpes)	n=368 (50.6)*
c) No risk situation (<0.1% risk of developing neonatal herpes)	n=97 (13.3)
d) Don't know	n=134 (18.4)

16) Can the patient breastfeed her child? (*: correct answer)

a) Yes	n=71 (98.2)*
b) No	n=13 (1.8)

Clinical vignette n°4 (question 17 to 20):

A 30-year-old nulliparous woman comes to the emergency room with vulvar pain for several days. She has no other symptoms and her pregnancy is proceeding normally. She is at the end of 25 weeks of gestation today.

On clinical examination, you find clusters of painful and inflammatory vesicles on the vulva and vagina, typical of a herpetic infection. You ask the patient about her history and she tells you that she has had pimples regularly for several years.

You have performed para-clinical examinations and the laboratory gives you the results:

PCR HSV1+ / PCR HSV2 - / IgG HSV1- / IgG HSV 2 +

17) What do you think is the most likely diagnosis? (*: correct answer)

a) Initial primary infection	n=84 (11.5)
b) Initial non-primary infection	n=415 (57.0)*
c) Recurrence	n=192 (26.4)
d) I don't know	n=37 (5.1)

18) What do you think is the appropriate treatment for this pregnancy? (*: correct answer)

- No treatment	n=2 (0.2)
- Prophylactic antiviral treatment at 36 weeks of gestation	n=510 (70.1)*
- Therapeutic antiviral treatment	n=661 (90.8)*

18bis) What do you think is the appropriate management for this pregnancy? (*: correct answer)

- Programmed cesarean section at term	n=13 (1.8)
- vaginal delivery at term if no new herpetic manifestations	n=580 (79.7)*
- Don't know	n=135 (18.5)

After appropriate management and a pregnancy without recurrence of herpes, the parents are worried about the risk to their child of neonatal herpes. They talk to you and discuss the herpetic episode at 25 days' gestation.

19) What can you say about the risk of neonatal herpes in this asymptomatic newborn? (*: correct answer)

a) High risk situation (>20% risk of developing neonatal herpes)	n=14 (1.9)
b) Low risk situation (<20% risk of developing neonatal herpes)	n=354 (48.6)*
c) No risk situation (<0.1% risk of developing neonatal herpes)	n=233 (32.0)*
d) Don't know	n=127 (17.5)

20) Can the patient breastfeed her child? (*: correct answer)

a) Yes	n=726 (99.7)*
b) No	n=2 (0.3)

Clinical vignette #5 (questions 21-24):

A 30-year-old nulliparous woman comes to you for her pregnancy consultation. She does not describe any symptoms and her pregnancy is proceeding normally. She is at the end of 25 weeks of gestation on this day.

On clinical examination, you find clusters of vesicles on the vulva and vagina, typical of a herpes infection. However, these vesicles are not felt by the patient. You ask the patient about her history and she tells you that she has had these pimples regularly for several years.

You have performed para-clinical tests and the laboratory gives you the results:

PCR HSV1 + / PCR HSV2 - / IgG HSV1 + / IgG HSV2 -

21) What do you think is the most likely diagnosis? (*: correct answer)

a) Initial primary infection	n=18 (2.5)
b) Initial non-primary infection	n=47 (6.5)
c) Recurrence	n=647 (88.8)*
d) Don't know	n=16 (2.2)

22) What do you think is the appropriate treatment for this pregnancy? (*: correct answer)

- No treatment	n=33 (4.5)
- Prophylactic antiviral treatment at 36 weeks of gestation	n=531 (72.9)*
- Therapeutic antiviral treatment	n=547 (75.1)*

22bis) What do you think is the appropriate management for this pregnancy? (*: correct answer)

- Programmed cesarean section at term	n=12 (1.7)
- vaginal delivery at term if no new herpetic manifestations	n=581 (79.8)*
- Don't know	n=135 (18.5)

After appropriate management and a pregnancy without recurrence of herpes, the parents are concerned about the risk to their child and the severity associated with neonatal herpes. They talk to you and discuss the herpetic episode at 25 days' gestation.

23) What can you say about the risk of neonatal herpes in this asymptomatic newborn? (*: correct answer)

a) High risk situation (>20% risk of developing neonatal herpes)	n=10 (1.4)
b) Low risk situation (<20% risk of developing neonatal herpes)	n=253 (34.8)*
c) No risk situation (<0.1% risk of developing neonatal herpes)	n=364 (50.0)*
d) Don't know	n=101 (13.8)

24) Can the patient breastfeed her child? (*: correct answer)

a) Yes n=723 (99.3)*

b) No n=5 (0.7)

Clinical vignette n°6 (question 25 to 28):

A 30-year-old nulliparous woman comes to the emergency room with vulvar pain for several days. She has no other symptoms and her pregnancy is proceeding normally. She is at 38 weeks of gestation today.

On clinical examination, you find clusters of painful and inflammatory vesicles on the vulva and vagina, typical of a herpetic infection. You ask the patient about this history and she tells you that she has never had these symptoms before.

You have performed para-clinical examinations and the laboratory gives you the results:

PCR HSV1 + / PCR HSV2 - / IgG HSV1 - / IgG HSV2 -

25) What do you think is the most likely diagnosis? (*: correct answer)

a) Initial primary infection n=708 (97.2)*

b) Initial non-primary infection n=8 (1.2)

c) Recurrence n=3 (0.4)

d) Don't know n=9 (1.2)

26) What do you think is the appropriate treatment for this pregnancy? (*: correct answer)

- No treatment n=5 (0.6)
- Prophylactic antiviral treatment at 36 weeks of gestation n=278 (38.2)
- Therapeutic antiviral treatment n=555 (76.2)*

26bis) What do you think is the appropriate management for this pregnancy? (*: correct answer)

- Programmed cesarean section at term n=364 (50.0)*
- vaginal delivery this day n=9 (1.2)
- Don't know n=355 (48.8)

While in the hospital, the parents are anxious about the risk to their child of neonatal herpes. They talk to you and discuss the herpes episode of a few days ago.

27) What can you say about the risk of neonatal herpes in this asymptomatic newborn? (*: correct answer)

a) High risk situation (>20% risk of developing neonatal herpes) n=374 (51.4)*

b) Low risk situation (<20% risk of developing neonatal herpes) n=207 (28.4)

c) No risk situation (<0.1% risk of developing neonatal herpes) n=39 (5.4)

d) Don't know n=108 (14.8)

28) Can the patient breastfeed her child? (*: correct answer)

a) Yes n=704 (96.7)*

b) No n=24 (3.3)

French guidelines

29) Do you know of recent French guidelines concerning herpes in pregnant women?

a) Yes

b) No

Table S2: Influence of knowledge of guidelines on practices

Type of maternal infection	Number (percentage) of answers according to the term of pregnancy.					
**Initial primary infection**	**25 weeks of gestation**			**38 weeks of gestation**		
**Diagnosis**	**Correct** (n=693)	*p-value*	*adjusted p-value*	**Correct** (n=708)	*p-value*	*adjusted p-value**
Know guidelines	187 (98.4%)	**p = 0.026**	p = 0.059	186 (97.9%)	p = 0.710	p = 0.651
Don't know guidelines	506 (94.1%)			522 (97%)		
**Care***	**Correct** (n=364)			**Correct** (n=254)		
Know guidelines	121 (63.7%)	**P<0.0001**	**P<0.0001**	75 (39.5%)	p = 0.146	p = 0.129
Don't know guidelines	242 (45.2%)			179 (33.3%)		
**Neonatal risk**	**Good Evaluation** (n=614)			**Good Evaluation** (n=374)		
Know guidelines	164 (86.3%)	p = 0.450	p = 0.448	103 (54.2%)	p = 0.410	p = 0.356
Don't know guidelines	450 (83.6%)			271 (50.4%)		
**Breastfeeding**	**Possible** (n=727)			**Possible** (n=704)		
Know guidelines	190 (100.0%)	p = 1	p = 0.982	188 (98.9%)	p = 0.075	p = 0.058
Don't know guidelines	537 (99.8%)			516 (95.9%)		
**Initial non-primary infection**	**25 weeks of gestation**			**38 weeks of gestation**		
**Diagnosis**	**Correct** (n=415)			**Correct** (n=345)		
Know guidelines	122 (64.2%)	**p = 0.025**	p = 0.11	108 (56.8%)	**p = 0.003**	**p = 0.005**
Don't know guidelines	293 (54.5%)			237 (44.1%)		
**Care***	**Correct** (n=392)			**Correct** (n=175)		
Know guidelines	131 (68.9%)	**p <0.0001**	**p <0.0001**	58 (30.5%)	**p = 0.019**	**p = 0.027**
Don't know guidelines	261 (48.5%)			117 (21.7%)		
**Neonatal risk**	**Good Evaluation** (n=587)			**Good Evaluation** (n=290)		
Know guidelines	164 (86.3%)	**p = 0.03**	**p = 0.045**	84 (44.2%)	p = 0.180	p = 0.180
Don't know guidelines	423 (78.6%)			206 (38.3%)		
**Breastfeeding**	**Possible** (n=726)			**Possible** (n=714)		
Know guidelines	189 (99.5%)	p = 1.000	p = 0.240	189 (99.5%)	p = 0.130	p = 0.130
Don't know guidelines	537 (99.8%)			525 (97.6%)		
**Recurrence**	**25 weeks of gestation**			**38 weeks of gestation**		
**Diagnosis**	**Correct** (n=647)			**Correct** (n=659)		
Know guidelines	173 (91.1%)	p = 0.330	p = 0.680	175 (92.1%)	p = 0.470	p = 0.420
Don't know guidelines	474 (88.1%)			484 (90.0%)		
**Care***	**Correct** (n=436)			**Correct** (n=133)		
Know guidelines	135 (71.1%)	**p < 0.0005**	**p < 0.005**	40 (21.1%)	p = 0.300	p = 0.240
Don't know guidelines	301 (55.9%)			93 (17.3%)		
**Neonatal risk**	**Good Evaluation** (n=617)			**Good Evaluation** (n=368)		
Know guidelines	167 (87.9%)	p = 0.200	p = 0.390	99 (52.1%)	p = 0.680	p = 0.710
Don't know guidelines	450 (83.6%)			269 (50.0%)		
**Breastfeeding**	**Possible** (n=723)			**Possible** (n=715)		
Know guidelines	190 (100.0%)	p =0.320	p = 0.980	189 (99.5%)	p = 0.200	p = 0.150
Don't know guidelines	533 (99.1%)			526 (97.8%)		

* care = treatment and mode of birth
